# Do you look longer at attractive faces? It depends on what you are looking for

**DOI:** 10.1177/20416695241286413

**Published:** 2024-10-13

**Authors:** Dirk Kerzel, Nicolas Prigoda, Olivier Renaud

**Affiliations:** Department of Psychology, 27212 University of Geneva, Genève, Switzerland

**Keywords:** visual search, evolutionary psychology, attractiveness, attentional bias

## Abstract

Evolutionary psychology suggests that we are attuned to relevant information in the environment. For example, attention may be attracted by physical beauty because it is important for finding a partner with good reproductive health. Consistently, previous studies found that attention stayed longer on attractive than unattractive faces. We asked whether this tendency was automatic and varied participants’ implicit search intentions to be either consistent or inconsistent with the presumably automatic tendency to attend to attractive faces. To create an implicit intention to look at attractive faces, participants searched for a happy face in an array of neutral faces because happy faces are rated as more attractive than neutral faces. To create the opposite intention to look at unattractive faces, participants searched for a disgusted or sad face because disgusted or sad faces are rated as less attractive than neutral faces. We found longer fixation durations on attractive faces when participants searched for happy faces. When participants searched for disgusted or sad faces, however, fixation durations were longer on unattractive faces. Thus, the search task determined whether attractive faces were looked at longer. The tendency to attend to attractive faces is therefore not automatic but can be overruled by search intentions.

It is a central idea of evolutionary psychology that humans are selectively attuned to adaptively relevant features of the environment (Buss, 2019). For instance, humans are thought to prefer physical attractiveness because it may indicate (reproductive) health (Grammer et al., 2003; Thornhill & Gangestad, 1999). Another reason may be that looking at physical beauty has reward-like effects (Aharon et al., 2001). A case in point for the evolutionary perspective is that there is a tendency to look longer at attractive faces. Longer fixation durations on attractive faces either suggest that attractive faces capture attention in an automatic manner or they suggest that participants voluntarily attend to attractive faces. In general, automatic control of attention reflects characteristics of the stimulus whereas voluntary control of attention reflects requirements of the task (Jonides, 1981; Liesefeld et al., 2024). In the present study, we assessed whether attention to attractive faces is automatic or voluntary. Before we talk about studies on overt attention where the duration of eye fixations was measured as in the current study, we review the literature on covert attention where eye movements were avoided.

Sui and Liu (2009) suggested that facial beauty automatically competes with the current task for attention. In their study, an endogenous cue at central fixation preceded a target letter on either side of fixation. An attractive or unattractive face was presented at the same time as the target, but on the opposite side of fixation. Reaction times (RTs) were shorter when the endogenous cue indicated the target location correctly, confirming that participants had shifted attention to the side indicated by the endogenous cue. On these trials, RTs were longer when the face on the other side was attractive than when it was unattractive. Because the face was task-irrelevant and appeared on the unattended side, the prolonged RTs suggest that processing of the attractive face competed automatically with the letter discrimination task. The competition from attractive faces was stronger than from unattractive faces even when the face did not reach conscious awareness (Hung et al., 2016). However, the study of Sui and Liu (2009) does not clarify how exactly the attractive faces interfered with the ongoing task. Roughly, participants were paying more attention to attractive than unattractive faces, which may also explain why change detection in an array of faces was worse when all faces were attractive (Chen et al., 2012). However, other studies investigated the attentional mechanisms more precisely and suggested that it may be the disengagement of attention that is slower for attractive than for unattractive faces. For instance, Maner et al. (2007) observed in a dot-probe task that it took longer to respond to a target on one side of fixation when it was preceded by an attractive face on the opposite side of fixation, suggesting that the disengagement of attention from the face was delayed with attractive compared to unattractive faces. Similarly, Valuch et al. (2015) showed that saccades to a peripheral target were more strongly delayed when an attractive compared to an unattractive face appeared at central fixation. Thus, it appears that it is more difficult to disengage covert attention from attractive than unattractive faces.

Consistent with delayed disengagement of covert attention, fixation durations under free-viewing conditions were found to be longer for attractive than unattractive faces (Goller et al., 2019; Leder et al., 2010, 2016; Maner et al., 2003; Mitrovic et al., 2018). For instance, participants in Leder et al. (2016) were shown images of real-world scenes with frontal views of two individuals. They were instructed to freely view the images for 10 s without any task. Eye movement recordings revealed that participants looked longer at individuals who were rated as more attractive. Thus, studies on covert and overt attention converge on the conclusion that the allocation of attention increases with increasing attractiveness. Attractive faces slow RTs when they are distractors, presumably because the disengagement of attention is delayed, or they are looked at longer when there is no experimental task.

In the current contribution, we evaluated whether the tendency to attend longer to attractive than unattractive faces is automatic in the sense that it overrules the current intentions of the participant, or whether the current intentions of the participant overrule the tendency to look at attractive faces. In contrast to previous studies, we will employ a visual search task because visual search is a frequent activity in everyday life (Wolfe, 2021) and is therefore more ecologically valid than tasks requiring central fixation. Further, search tasks involve the intention to find a particular target which enables us to control the task-relevant features. Thus, the research question is novel because previous studies either did not involve eye movements (Chen et al., 2012; Hung et al., 2016; Maner et al., 2007; Sui & Liu, 2009) or did not use a visual search task (Goller et al., 2019; Leder et al., 2010, 2016; Maner et al., 2003; Mitrovic et al., 2018; Silva et al., 2016; Valuch et al., 2015). Automatic versus voluntary deployment of attention has already been examined in the context of spatial cueing studies where some concluded that spatial cues were attended even if it was better to attend away from them, suggesting that effects of spatial cues are automatic (Driver et al., 1999; Warner et al., 1990).

The role of search intentions has been amply described in the discussion of top-down versus bottom-up control of attention (Liesefeld et al., 2024). On the one hand, current search intentions determine in a top-down manner which features capture attention. For instance, when looking for a red target, red cues capture attention, whereas green cues do not. When looking for a green target, however, it is the other way around (Folk & Remington, 1998). On the other hand, there is also evidence that salient stimuli capture attention irrespective of current search intentions. For instance, a salient color singleton may capture attention when participants search for a shape target, suggesting that saliency overrules top-down search intentions (Luck et al., 2021).

## Experiment 1

**Figure 1.**
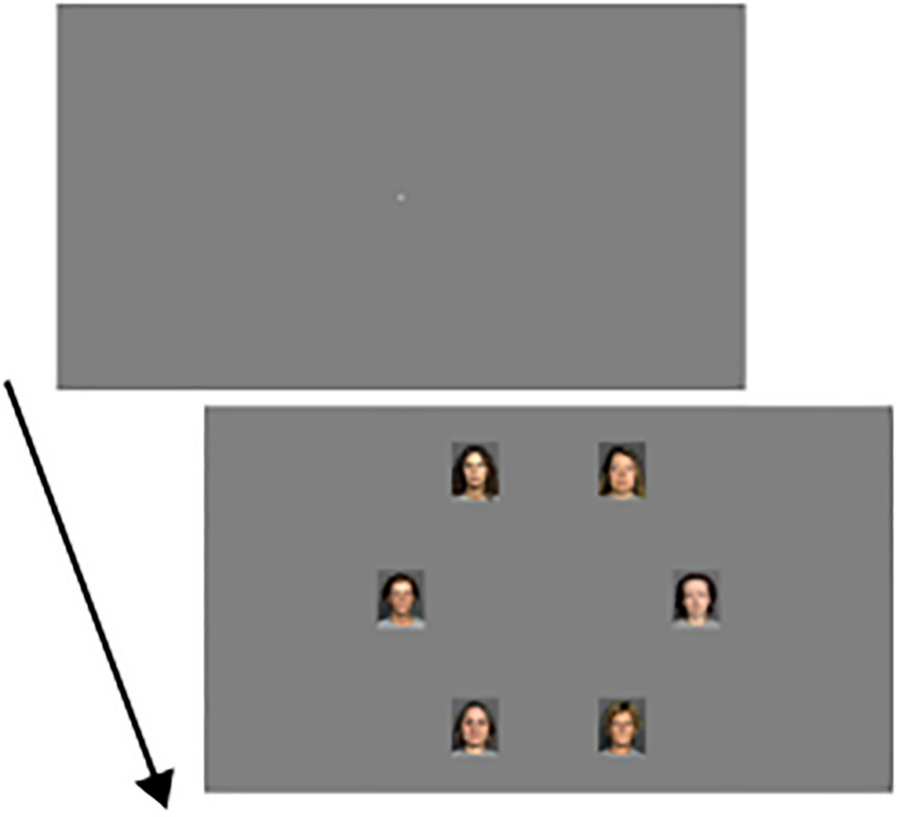
Illustration of experimental stimuli in Experiments 1 and 2. The figure shows miniatures of the screen that would extend 48.4 cm horizontally and 30.2 cm vertically in the experiments. For sample images, please consult http://faces.mpib-Berlin.mpg.de. The fixation display was shown for 1,000 ms before the search display appeared.

We asked whether search intentions that conflict with the supposedly automatic tendency to look at attractive faces can overrule this tendency. In Experiment 1, we induced an implicit intention to look for unattractive faces by asking participants to find a face showing either a sad or disgusted expression among faces with neutral faces (see Figure 1). Disgusted and sad faces are evaluated as less attractive than neutral faces (see p. 9 and Figure 3A in Ebner et al., 2018; Mueser et al., 1984). Therefore, the intention to find a sad or disgusted expression may induce a tendency to look longer at unattractive neutral faces. Note that this intention is implicit as participants were not instructed to look for unattractive faces, but to look for a sad or disgusted expression.

### Methods

#### Transparency and Openness Promotion

To determine the number of participants, we relied on previous studies using free-viewing tasks, which had sample sizes between 30–57 participants (Goller et al., 2019; Leder et al., 2016). To control for possible effects of gender, we collected data from as many men as women. Thirty-two first-year psychology students participated for class credit and reported normal or corrected-to-normal vision (16 male; age: *M *= 21 years, *SD *= 2, range 19–25 years). The study was approved by the ethics committee of the Faculty of Psychology and Educational Sciences and was carried out in accordance with the Code of Ethics of the World Medical Association (Declaration of Helsinki). Informed written consent was given before the experiment started. Further, the authors declare that they have no conflict of interest. None of the experiments reported in this article was formally preregistered. The data as well as the R-script for the analysis of fixation durations are available on the Open Science Framework (https://osf.io/g32mv). The code for running the experiment is available on request. Data were collected in 2023.

#### Apparatus

The stimuli were displayed on a 22.5-in. liquid-crystal display monitor (100 Hz, 1,200 × 1,200 pixels, VIEWPixx Lite, standard backlight, VPixx Technologies Inc., Saint-Bruno, Canada). Head position was stabilized with a chin/forehead rest at a viewing distance of 66 cm. The left and right buttons on a RESPONSEPixx Handheld five-button response box (VPixx Technologies Inc., Saint-Bruno, Canada) were used to judge the facial expression. The experiment was run using PsychoPy2 (Peirce et al., 2019) on an IBM-compatible PC. A desktop-mounted EyeLink1000 (SR Research, Ontario, Canada) was used to record eye movements at a sampling rate of 1000 Hz. To detect saccades, we set the EyeLink1000 to the standard saccade criteria for cognitive research (i.e., velocity of 30°/s and acceleration of 8,000°/sec^2^).

#### Stimuli

In the fixation display, the fixation cross (0.24° diameter) was shown in the center of an otherwise empty screen. The background was medium gray (43 cd/m^2^). In the search display, six images were shown on an imaginary circle where the distance between the fixation cross and the center of the images was 10°. The images were evenly distributed around the circle with two images on the horizontal meridian (see Figure 1). Each image was 2.9° wide and 3.6° high. Images were taken from the database presented in Ebner et al. (2018) which contains attractiveness ratings for images of male and female faces in three age ranges with several facial expressions. The images are in portrait style and show the model's hair. The background is dark gray, and parts of a light gray T-shirt worn by the models are visible. The images were downsized from 2,835 × 3,543 pixels (width × height) to 131 × 164 pixels using the Python imaging library in Python. The age ranges of the models were young (20–31 years), middle-aged (44–55 years), and older (70–81 years). The facial expressions were neutral, happy, sad, angry, fearful, and disgusted. For each combination of gender and age, there was a set of 27–29 different faces with neutral expression. On each trial, we selected six images from one of these sets. To have a wide range of attractiveness scores on each trial, we selected three faces among the twelve most attractive faces and another three among the twelve least attractive faces. The target was created by replacing one image with a neutral expression by another image of the same model with a sad or disgusted expression. Further, there were two versions for each facial expression and model. We averaged attractiveness across the two versions to select the images for display, but the version-specific attractiveness ratings were used in the data analysis. Attractiveness ratings were available from female and male raters in each of the three age ranges. We used the ratings from young raters matching the participants’ gender (i.e., from young male or female raters for male and female participants, respectively). Ratings and images can be downloaded from https://faces.mpdl.mpg.de/imeji/.

#### Design

All faces in the search display had the same gender and age range. The 72 combinations resulting from 2 (gender of faces in search display: male, female) × 3 (age range of faces in search display: young, middle-aged, old) × 2 (facial expression of target face: sad, disgusted) × 6 (location of target face) were presented twice in each trial block. Two trial blocks were run resulting in 288 trials per participant.

#### Procedure

At the start of a trial, the fixation cross was shown for 1,000 ms. Then, the program waited until eye gaze was within 1° of the fixation cross. If this did not occur within 5,000 ms, recalibration was initiated. Then, the fixation cross disappeared at the same time as the search display appeared. Participants were asked to search for the face with an emotional expression and press the left button for disgust and the right button for sad. Participants were instructed to respond as rapidly and accurately as possible. The experiment was preceded by 30 practice trials.

#### BFRT-r

After the experiment, we asked participants to complete the revised version of the Benton Facial Recognition Test (BFRT-r), which measures the ability to recognize faces (Murray et al., 2022). The scores of our participants ranged from 33 to 51 with *M *= 44.1, *SD *= 5.0. The maximal score is 54. We had expected a correlation between the ability to recognize faces and the time to find the emotional expression, but the correlation was not significant, Spearman's *r*(30) = .14, *p *= .438.

#### Analysis

Eye fixations were determined by the EyeLink parser and stored in EyeLink Data Format (EDF)-files, which were converted to a MatLab-compatible format using the Edf2Mat toolbox developed by Adrian Etter at the University of Zürich (see https://github.com/uzh/edf-converter). Fixations within 4° of the center of an image were assigned to the respective face. The distance between adjacent image centers was 10°.

### Results

#### Total Fixation Duration per Image

**Figure 2.**
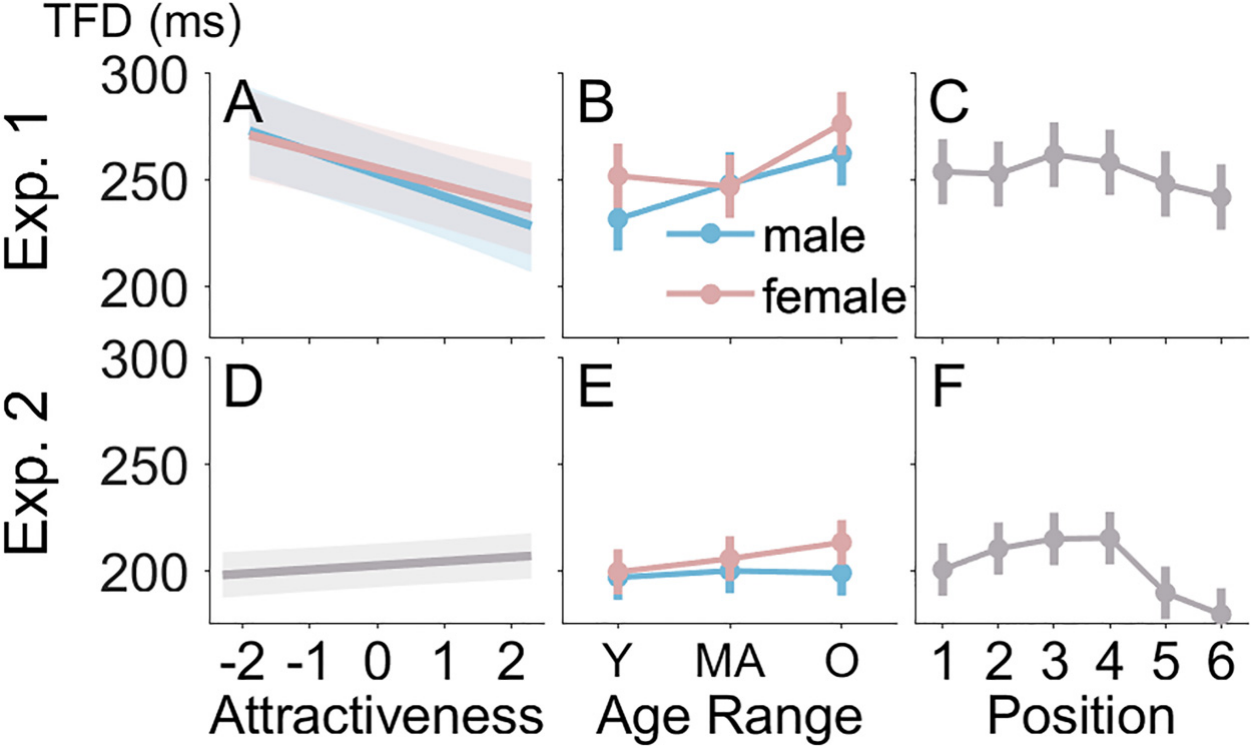
Total fixation durations in Experiments 1 and 2. TFDs in milliseconds are shown on the *y*-axis. Panels A, B, and C show results from Experiment 1, and Panels D, E, and F show results from Experiment 2. Plots in Panels A and D show TFDs as a function the *z*-transformed attractiveness rating. Plots in Panels B and E show the interaction of age range and gender of the fixated face. Plots in Panels C and F show effects of the fixated face's position on TFDs. In Panel A, “male” and “female” refer to the gender of the participant, whereas in Panels B and E, it is the gender of the fixated face. Error bars represent the 95% confidence interval. TFD = total fixation duration; Y = young; MA = middle-aged; O = older; 1 = right; 2 = upper right; 3 = upper left; 4 = left; 5 = lower left; 6 = lower right.

On average, participants made 7.1 fixations per trial and 96% of these fixations could be assigned to one of the faces. The target face was looked at on 97% of trials and at least one nontarget face was looked at on 93% of trials. On the latter trials, an average of 3.6 nontarget faces were fixated. To calculate total fixation time, we summed across refixations of the same face. Because not all faces were looked at and attractiveness ratings were continuous, conventional analysis of variance (ANOVA) models could not be applied. Instead, we used linear mixed models (LMMs). Before running the LMM, we *z*-transformed the attractiveness ratings and excluded fixations on the target face. Another 17 fixations were excluded because the RT on the respective trial was longer than 10 s, leaving 30’534 fixations for analysis. We ran the LMM using the lme4 package (Bates et al., 2015) and lmerTest (Kuznetsova et al., 2017) in R Version 4.3.1 (R Core Team, 2021). We therefore report degrees of freedom according to Satterthwaite's correction and the corresponding *p*-values. We included the following factors in the LMM: the *z*-transformed attractiveness rating of the fixated face, the interaction of *z*-transformed attractiveness rating with gender of the participant, the main effects and interaction of gender and age range of the fixated face, and the position of the fixated face. The equation of the model was FixationDuration ∼ FaceAttractiveness×ParticipantGender + FaceGender×FaceAge + FacePosition + (1|participant) + (1|participant:faceGender:faceAge) + (1|participant:facePosition) + (faceAttractiveness −1|participant). As advocated by Matuschek et al. (2017), we did include the random “slopes” corresponding to fixed effects, while keeping a model where optimization converges. As recommended by Shatz (2024), we checked the assumption of normality in the LMM using diagnostic tools instead of testing them. The diagnostic tools on the final model were satisfactory. In addition, using the logarithm of the duration led to highly similar results (same significant effects and very similar significance) and quite satisfactory diagnostic plots, showing the robustness of the results. However, we provide the results from the raw duration as these values are easier to interpret. The mean total fixation durations are shown in Figure 2. Most importantly, the LMM revealed that total fixation duration per face decreased as the attractiveness of the face increased (see Figure 2A), *F*(1, 42.9) = 39.07, *p *< .001. That is, less attractive faces were looked at longer, which is opposite to the supposedly automatic tendency to look at attractive faces but corresponds to the implicit bias induced by the task. The effect of attractiveness rating was not further qualified by gender of the participant, *p *= .393, suggesting that the effect of attractiveness rating was similar for male and female participants. Effects of gender of the fixated face, *F*(1, 166.5) = 17.40, *p *< .001, and age range of the fixated face, *F*(2, 181.7) = 36.55, *p *< .001, entered an interaction, *F*(2, 152.5) = 6.01, *p *= .003. Inspection of Figure 2B shows that the total fixation duration per fixated face increased with the age range of the face and was shorter for male than female faces except for middle-aged faces, where fixation durations were similar for male and female faces. Finally, the position of the fixated face affected fixation duration and was longest when the face appeared in the upper left or left position (see Figure 2C), *F*(5, 153.3) = 3.49, *p *= .005, which may be related to a leftward bias in viewing behavior (Ossandón et al., 2014). The gender of the participant did not affect fixation durations, *p *= .859.

#### First Fixations

In addition to total fixation times, we analyzed the location of the first fixation after onset of the images (e.g., Fletcher-Watson et al., 2008; Thompson et al., 2019). First, we evaluated whether first saccades were automatically directed to attractive faces in the periphery. We analyzed saccades with latencies longer than 50 ms that went to one of the nontarget faces (79% of trials). The average median latency of the first fixation was 197 ms (*SD *= 28 ms). To evaluate whether the face that was fixated first was more attractive than the others, we ranked the attractiveness of the five nontarget faces and determined the rank of the face that was fixated first. The mean rank of this face was 3.0 (rounded, *SD *= 0.1), which was not significantly different from 3.0, *t*(31) = 1.01, *p *= .322, Cohen's *d_z _*= 0.10. Because a rank of three among five is expected with randomly directed saccades, this result suggests that first saccades were not guided toward more attractive faces. Second, we evaluated whether participants were able to locate the emotional face in the periphery while looking at the central fixation cross at the beginning of the trial. First saccades were directed at the emotional face in 19% of trials, which is significantly different from chance (1/6 or 17%), *t*(31) = 4.07, *p *< .001, Cohen's *d_z _*= 0.72. While statistically different from chance, the ability to locate the emotional expression was poor. The vast majority of first saccades (81%) were directed at nontarget faces without emotional expression. These results are consistent with the conclusion that emotional facial expressions do not guide attention (Frischen et al., 2008; Wolfe & Horowitz, 2004), but also with the hypothesis that the eccentricity of the emotional face (10°) was too large to allow for useful analysis of its features.

#### Search RTs

**Figure 3.**
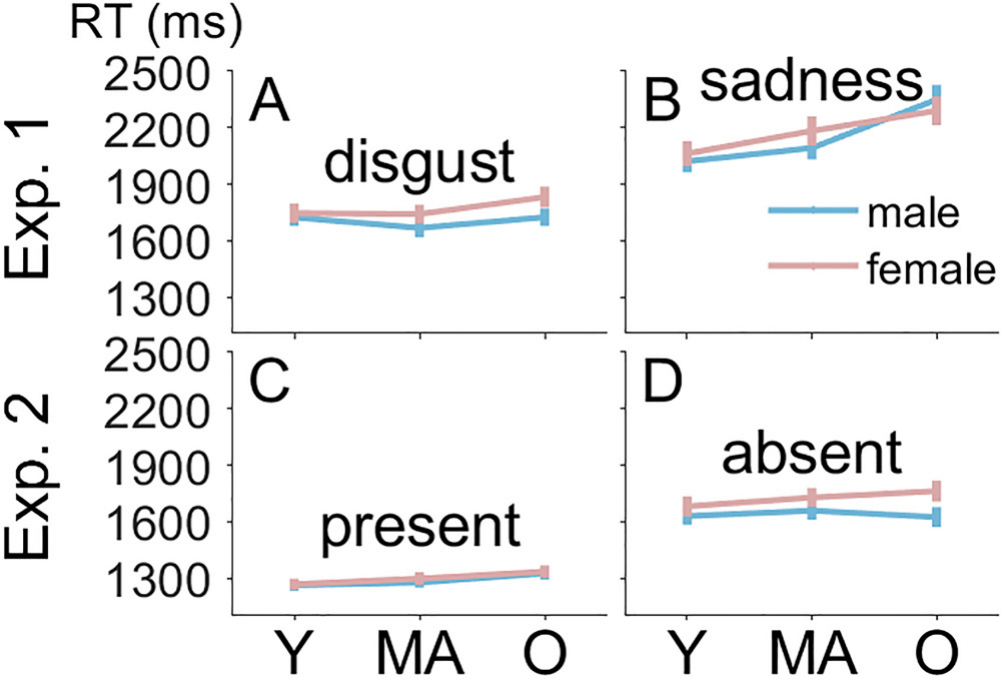
Search reaction times in Experiments 1 and 2. Search RTs in milliseconds are shown on the *y*-axis. Panels A and B show results from Experiment 1, and Panels C and D show results from Experiment 2. Panel A shows results for the disgusted target expression, and Panel B shows results for the sad target expression. Panel C shows results for trials where the happy target face was present, and Panel D shows results for trials where it was absent. Error bars represent the between-participant standard error of the mean. RT = reaction time; Y = young; MA = middle-aged; O = older.

We analyzed search RTs and search errors for completeness, but these results do not speak directly to our experimental hypothesis. Among other things, search RTs reflect decision processes once the emotional expression was fixated, but these fixations were excluded in the analysis of fixation durations presented above. We applied conventional ANOVA models because individual medians could be calculated for each cell of the experimental design. Before doing so, we excluded 13% of trials with choice errors. The means of individual median RTs are shown in Figure 3A and 3B. We conducted a 2 (facial expression of target face: disgust, sadness) × 3 (age range of faces: young, middle-aged, older) × 2 (gender of faces: male, female) within-participant ANOVA. RTs were shorter with disgust than sadness (1,739 vs. 2,167 ms), *F*(1, 31) = 141.30, *p *< .001, η_p_^2 ^= .82, increased with age range of the face (1,889, 1,921, 2,049 ms), *F*(2, 62) = 28.54, *p *< .001, η_p_^2 ^= .48, and tended to be shorter for male than female faces (1,930 vs. 1,976 ms), *F*(1, 31) = 4.01, *p *= .054, η_p_^2 ^= .11. The interaction of facial expression and age range, *F*(2, 62) = 11.89, *p *< .001, η_p_^2 ^= .28, showed that the increase with age range was stronger for sadness (2,042, 2,138, 2,320 ms; Figure 3B) than for disgust (1,735, 1,705, 1,778 ms, Figure 3A). These results were replicated using the logarithm of RT to improve the normality of the data.

**Figure 4.**
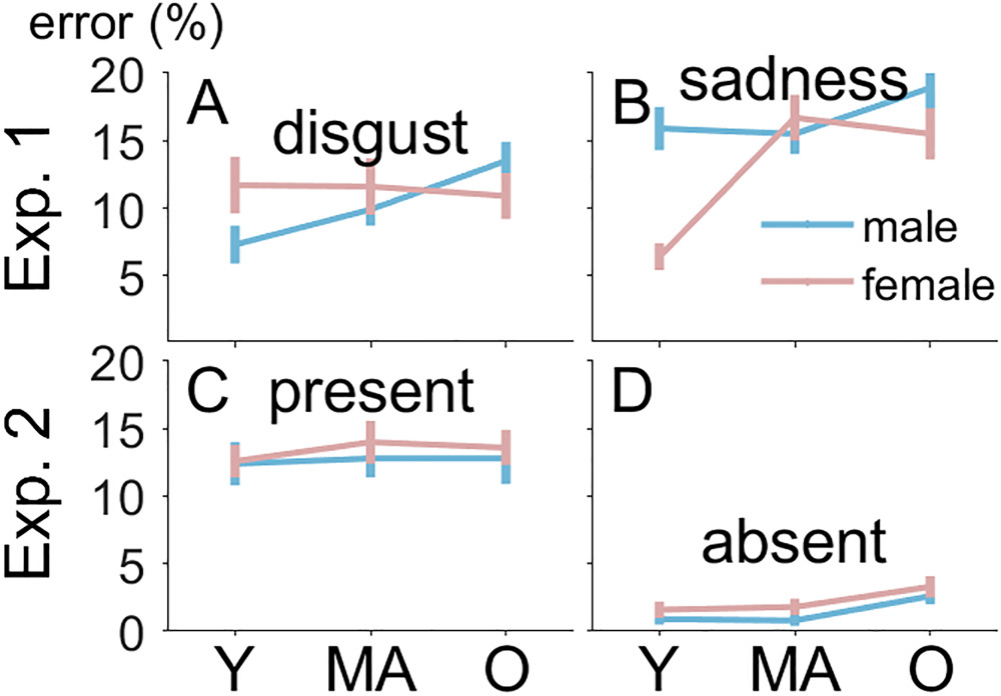
Error percentages in Experiments 1 and 2. Error percentages are shown on the *y*-axis. Panels A and B show results from Experiment 1, and Panels C and D show results from Experiment 2. Error bars represent the between-participant standard error of the mean. RT = reaction time; Y = young; MA = middle-aged; O = older.

The analysis of choice errors confirmed some results of the RT analysis, but also showed evidence for facilitated emotion discrimination in some sets of images. As can be seen in Figure 4A and 4B, error percentages were lower for disgust than sadness (11% vs. 15%), *F*(1, 31) = 10.13, *p *= .003, η_p_^2 ^= .25, and increased with age (10%, 13%, 15%), *F*(2, 62) = 11.80, *p *< .001, η_p_^2 ^= .28, which reflects the RT data. The two-way interactions of face gender with facial expression, *F*(1, 31) = 10.22, *p *= .003, η_p_^2 ^= .25, and face gender with age range of the face, *F*(1, 31) = 3.63, *p *= .032, η_p_^2 ^= .11, were modulated in a three-way interaction of facial expression, gender of face, and age range of face, *F*(1, 31) = 10.35, *p *< .001, η_p_^2 ^= .25, which was not observed in the RT data. Generally, error percentages increased more strongly with age range for male than female faces, but there was one exception. Young female faces with a sad expression produced lower error percentages than other sad faces (6% vs. more than 15%; see Figure 4B), suggesting that discrimination between disgust and sadness was facilitated in this set of faces. These effects were confirmed using arcsine-transformed error proportions to homogenize variances, except for the interaction of face gender and age range, *p *= .081.

### Discussion

We asked participants to look for an emotional facial expression in an array of neutral faces and to indicate whether the emotion was disgust or sadness. Because disgusted or sad faces are perceived as less attractive, the task created the implicit intention to look for unattractive faces. Consistently, we observed that fixation durations on neutral faces were longer for unattractive than attractive faces, which is inconsistent with the idea that fixation times increase automatically for attractive faces. Rather, the implicit search intentions overruled the tendency to look at attractive faces, showing that the tendency is not automatic. Further, we found that the effect of attractiveness was the same for male and female participants, unlike in some previous reports (Maner et al., 2003; Mitrovic et al., 2018; Valuch et al., 2015; van Hooff et al., 2011). However, effects of gender were not the focus of the study. Possibly, we did not have enough power to detect these effects, but we do not know for sure. The reason is that power analyses for linear mixed effect models require selection of reasonable values for every parameter in the model. As the model in Experiment 1 contained 13 parameters and selection of these values is not straightforward, we could not perform a power analysis to clarify this issue.

## Experiment 2

Experiment 1 showed that the instruction to look for sad or disgusted faces induced a tendency to look longer at unattractive faces. To provide further evidence that search intentions determine the relation between fixation duration and attractiveness, we used the same stimulus set as in Experiment 1 but changed the task. That is, we asked participants to indicate whether a happy face was present or absent in the search display. Previous research showed that happy expressions receive higher attractiveness ratings than neutral expressions (see p. 9 and Figure 3A in Ebner et al., 2018; O’Doherty et al., 2003; Otta et al., 1996). Therefore, the intention to find a happy face may induce an implicit intention to look for attractive faces, which is expected to increase fixation durations on attractive faces. We switched from an emotion discrimination task with two emotional expressions to a detection task with a single emotional expression because only happy expressions were rated as more attractive than neutral expressions (Ebner et al., 2018). Sad, angry, fearful, and disgusted were all rated as less attractive.

### Methods

Because gender of the participant did not interact with face attractiveness in Experiment 1, we did not aim for the same number of male and female participants in Experiment 2. Forty first-year psychology students participated for class credit and reported normal or corrected-to-normal vision, but only 37 were retained in the final analysis (three male; age: *M *= 21 years, *SD *= 5, age range 17–40). Somewhat unexpectedly, there were two 40-year-old participants in our sample of first-year psychology students. The age range of the remaining participants was 17–26, which is close to the age range of the young raters (20–31 years) in Ebner et al. (2018) whose ratings were used to in the current study. Although the 40-year-old participants were closer to the age range of Ebner et al.'s middle-aged raters (44–55 years), we decided to keep them in the sample because removing their datasets did not change the results. Further, we excluded datasets when the participant's values were beyond 3 *SD* of the mean value in the remaining sample. That is, two datasets were excluded because only 75–77% of the eye fixations could be assigned to one of the faces (vs. *M *= 94%, *SD *= 5 in the rest of the sample). Another dataset was excluded because overall RTs were very slow (2,810 ms vs. *M *= 1,468 ms, *SD *= 226 in the rest of the sample).

The methods were as in Experiment 1, but the task was changed. Instead of discriminating between disgust and sadness, participants had to indicate whether a face with a happy expression was present or absent. That is, on target-present trials, one image with a neutral expression was exchanged for an image of the same model with a happy expression. On target-absent trials, only neutral expressions were shown. The 72 combinations resulting from 2 (gender of faces in search display: male, female) × 3 (age range of faces in search display: young, middle-aged, old) × 2 (happy face: present, absent) × 6 (location of happy face, applicable only on present trials) were presented twice in each trial block. Two trial blocks were run resulting in 288 trials per participant. The BFRT-r was not administered.

### Results

#### Total Fixation Duration per Image

On average, there were 6.1 fixations per trial and 94% of those fixations could be assigned to one of the faces. On 8% of target-present trials, no nontarget face was fixated, whereas this was true on only 0.3% of target-absent trials. Even after exclusion of these trials, we found that fewer nontarget faces were fixated on target-present than target-absent trials (3.1 vs. 5.5), *t*(36) = 5.65, *p *< .001, *d_z _*= 6.79. The average of 5.5 fixated faces on target-absent trials shows that participants performed an almost exhaustive search before deciding that the target was absent. Compared to the LMM conducted in Experiment 1, we added target presence and dropped gender of participant as factors. The model was FixationDuration ∼ FaceAttractiveness + FaceGender×FaceAge + FacePosition + HappyFacePresence + (1|participant) + (1|participant:faceAge) + (1|participant:faceGender:faceAge) + (1|participant:facePosition) + (1|participant:happyFacePresence) + (faceAttractivity-1|participant). We followed the same modeling strategy as in Experiment 1 and the diagnostic tools on the final model were satisfactory. There were 44’586 fixations available for analysis. The means are shown in Figure 2D to 2F. Most importantly, the effect of *z*-transformed attractiveness, *F*(1, 60.8) = 8.50, *p *= .005, showed that total fixation duration per face increased with attractiveness (see Figure 2D). That is, more attractive faces were looked at longer, which is opposite to the results from Experiment 1. Effects of gender of the fixated face, *F*(1, 133.8) = 47.93, *p *< .001, and age range of the fixated face, *F*(2, 87.5) = 12.41, *p *< .001, entered an interaction, *F*(2, 118.2) = 11.50, *p *< .001. Inspection of Figure 2E shows that the total fixation duration per image increased with the age range of the fixated face, but this effect was stronger for female than male faces. The position of the fixated face affected total fixation duration and was longest when it appeared in the upper left or left position and shorter when it was in the lower right or right position (see Figure 2F), *F*(5, 176.9) = 14.51, *p *< .001. Finally, fixation duration was longer on target-absent than target-present trials (210 vs. 188 ms), *F*(1, 36.1) = 33.50, *p *< .001.

#### Comparison between Experiments 1 and 2

To compare Experiments 1 and 2, we calculated individual averages before running independent-samples *t* tests. The number of faces fixated on target-present trials with at least one fixation on a nontarget face was significantly higher in Experiment 1 than 2 (3.6 vs. 3.1), *t*(67) = 5.19, *p *< .001, *d_s _*= 1.25. Also, the total fixation duration on these trials was longer in Experiment 1 than 2 (246 vs. 192 ms), *t*(67) = 6.11, *p *< .001, *d_s _*= 1.48. Consistent with the fixation data, the overall RTs were longer in Experiment 1 than in Experiment 2 (1,953 vs. 1,468 ms), *t*(67) = 8.08, *p *< .001, *d_s _*= 1.95, suggesting that the discrimination task in Experiment 1 was more difficult than the detection task in Experiment 2.

#### First Fixations

First fixations to a nontarget face with a minimal latency of 50 ms made up 38% of trials and had an average median latency of 206 ms (*SD *= 29 ms). We ranked the attractiveness of the five nontarget faces on each trial and determined the rank of the face that was fixated first. The mean rank of this face was 3.0 (rounded, *SD *= 0.1), which was not significantly different from 3.0, *t*(36) = 0.14, *p *= .893, Cohen's *d_z _*= 0.02, suggesting that attractiveness did not guide first fixations. Further, first saccades were directed at the happy face in 61% of trials, which is significantly different from chance (1/6 or 17%), *t*(31) = 92.9, *p *< .001, Cohen's *d_z _*= 15.28. Unlike in Experiment 1, participants were able to locate the emotional expression in the periphery and only a minority of first saccades (38%) were directed at nontarget faces first.

#### Manual Responses

For the analysis of RTs, we excluded 7% of trials because of choice errors. The means of individual medians are shown in Figure 3C and 3D. We conducted a 2 (happy target face: present, absent) × 3 (age range of faces: young, middle-aged, older) × 2 (gender of faces: male, female) within-participant ANOVA. RTs were shorter on trials where the target face was present than absent (1,280 vs. 1,657 ms), *F*(1, 36) = 225.29, *p *< .001, η_p_^2 ^= .86. RTs increased with age range (1,439, 1,474, 1,492 ms), *F*(2, 62) = 11.75, *p *< .001, η_p_^2 ^= .25, and were shorter for male than female faces (1,443 vs. 1,494 ms), *F*(1, 36) = 32.77, *p *< .001, η_p_^2 ^= .48. The interaction of target presence and gender, *F*(1, 36) = 11.07, *p *= .002, η_p_^2 ^= .24, showed that the difference between male and female faces was smaller on target-present (1,267 vs. 1,293 ms; see Figure 3C) than target-absent trial (1,619 vs. 1,695 ms; see Figure 3D). The interaction of target presence and age range of the faces, *F*(2, 72) = 3.96, *p *= .023, η_p_^2 ^= .10, showed that the increase with age range was stronger on target-present (1,245, 1,280, 1,314 ms) than target-absent trials (1,632, 1,668, 1,671 ms). These results were replicated using the logarithm of RT to improve the normality of the data, except for the last interaction, which only approached significance, *p *= .076.

The analysis of choice errors showed that error percentages were higher for present than absent responses (13 vs. 2%), *F*(1, 36) = 124.04, *p *< .001, η_p_^2 ^= .78. No other effect was significant, *p*s > .165. This effect was confirmed after arcsine transformation of the error proportions to homogenize variances. The means are shown in Figure 4C and 4D.

### Discussion

Participants were asked to search for a face with a happy expression in a detection task. Because the attractiveness of happy expressions is higher than the attractiveness of neutral expressions, the task induced an implicit intention to search for attractive faces. Consistently, we found that fixation durations increased with increasing attractiveness. That is, attractive faces were looked at longer. While we would like to chalk up this result to an implicit intention to look for attractive faces, it is also compatible with an automatic tendency to attend longer to attractive faces (e.g., Sui & Liu, 2009). Thus, Experiment 2 alone cannot decide between the effects of implicit search intentions and automatic tendencies but Experiments 1 and 2 together favor implicit search intentions because the effect of attractiveness was opposite in the two experiments. Another conclusion from Experiment 2 is that the inverse relation between fixation duration and attractiveness observed in Experiment 1 is not an artefact of the stimuli or the data analyses. Rather, the relation depends on the task and the associated search intentions. Further, search RTs, total fixation durations, and the number of fixations per face decreased in Experiment 2 compared to Experiment 1, suggesting that the task was easier. The reason may be that the teeth were visible in happy faces, which may have created a visual popout effect (Horstmann et al., 2012). In contrast, it is unlikely that distinctiveness explains the difference between Experiments 1 and 2 because all emotional expressions were judged to be more distinctive than neutral expressions and the distinctiveness of happy expressions was between disgusted and sad expressions (see p. 10 and Figure 3D in Ebner et al., 2018).

## General Discussion

We tested whether the tendency to look at attractive faces was automatic. Previous research found that attractive faces compete for attention even when participants focus their attention elsewhere (Sui & Liu, 2009). Therefore, the question arises whether the tendency to look longer at attractive faces (Goller et al., 2019; Leder et al., 2010, 2016; Maner et al., 2003; Mitrovic et al., 2018; Silva et al., 2016) is automatic. In Experiment 1, we employed a visual search task to pit the presumably automatic tendency to look at attractive faces against the implicit intention to look for unattractive faces. The implicit intention was created by asking participants to look for a disgusted or sad expression, both of which are judged to be less attractive than neutral expressions (see p. 9 and Figure 3A in Ebner et al., 2018). We found that fixation durations decreased with increasing facial attractiveness, which challenges the idea of an automatic tendency to look at attractive faces. In Experiment 2, we created an implicit intention to look for attractive faces by asking participants to indicate whether a happy face was present or absent. Happy faces were judged as more attractive than neutral faces (see p. 9 and Figure 3A in Ebner et al., 2018; O’Doherty et al., 2003; Otta et al., 1996). Consistently, fixation durations increased with attractiveness. Taken together, Experiments 1 and 2 provide evidence that (implicit) intentions determine whether attractive faces are looked at longer. In contrast, the idea of an automatic tendency to look at attractive faces predicts fixation durations to increase with attractiveness in all situations and fails to account for the results of Experiment 1.

### Search Intentions in Free Viewing Tasks

Thus, the current experiments suggest that there is no automatic tendency to look longer at attractive than unattractive faces. However, this conclusion does not question the validity of previous studies showing that participants prefer to look at attractive faces when there is no task associated with the faces (Goller et al., 2019; Leder et al., 2010, 2016; Maner et al., 2003; Mitrovic et al., 2018). These studies are different from the current task in many ways. For instance, the total fixation durations were much longer than in the present study. Here, total fixation durations were around 200 ms, whereas mean fixation durations were between 400 and 500 ms in previous studies with free viewing (Leder et al., 2010) and the total fixation duration was much longer (>6,000 ms in Goller et al., 2019). Most likely, the primary task in the current experiments incited participants to scan the images as rapidly as possible, leading to short fixation durations, which was not the case in free viewing tasks. Nonetheless, the brief fixation time was probably sufficient to appraise the beauty of the faces (Olson & Marshuetz, 2005). Further, in our experiments, the fixated images had to be compared to a stored representation of the target expressions “sad,” “disgusted,” or “happy” (see Eimer, 2014; Wolfe, 2021), whereas no such comparison was necessary in free viewing tasks. Therefore, the question arises why participants looked longer at attractive faces in free viewing tasks. Possibly, beauty corresponds to a default search intention that is activated when the system is idle. Otherwise, search for beauty is only activated when it is compatible with the task demands (e.g., when looking for happy faces). Thus, the attunement to relevant information in the environment (Buss, 2019) is not implemented as an automatic tendency to look longer at attractive faces, but rather as a default search intention that is activated in the absence of a search task. Longer fixation durations on attractive faces are therefore more compatible with top-down than bottom-up effects. The reason is that top-down or voluntary search intentions and not the bottom-up or stimulus-driven attractiveness of the faces determine fixation durations.

Our conclusion with respect to the different utilization of the same information for different tasks is consistent with a study on combined eye and mouse tracking by Faust et al. (2019). Faust et al. asked participants to perform a numerical judgment task while two irrelevant faces were shown close to the task-relevant numbers. The eye movements showed no difference between attractive and unattractive faces, suggesting that the numerical judgment task overruled the automatic tendency to look at attractive faces. In contrast, mouse movements deviated more strongly to attractive than unattractive faces, suggesting that different decision criteria were prioritized for eye and hand movements. This conclusion resonates with the current study which suggests that the prioritized information depends on task demands. Further, the current study allows for the reinterpretation of previous results. Nakamura and Kawabata (2014) found that the attentional blink was larger for attractive than unattractive faces. In this study, participants searched for two female faces in a stream of pictures with male faces. Consistent with the study of Sui and Liu (2009), it was argued that more attention was allocated to attractive than unattractive faces, resulting in a larger attentional blink. The present study suggests that another process may have contributed to this finding. As female faces are rated as more attractive than male faces, participants were looking for more attractive faces at the same time as they were looking for female faces. Consistent with this implicit search intention, more attention was allocated to attractive than unattractive target faces. Thus, implicit and explicit search intentions may have worked together to produce the results reported in Nakamura and Kawabata (2014).

### Distinctiveness and Effects of Gender and Age Range

In the current study, the total fixation durations and manual search responses were longer with female than male faces and increased with the age range of the face, suggesting that emotional expressions were harder to find in sets of female or older faces. The latter result is consistent with previous research showing that emotions are harder to read in older compared to younger faces (Ebner & Johnson, 2009; Riediger et al., 2011). Possibly, it was generally more difficult to find an emotional expression in sets with highly distinctive faces. Ebner et al. (2018) reported that female or older faces are judged as more distinctive than male or younger faces, respectively, which matches the longer fixation durations and search RTs for female and older faces. Similarly, Ebner et al. (2018) reported that disgusted expressions were judged as more distinctive than sad expressions, which matches the shorter search RTs for disgust than sadness. However, some of the results, particularly the three-way interaction in the analysis of error percentages in Experiment 1, are probably due to face-specific confusability or lack thereof in some sets of images. These may result from idiosyncratic facial expressions that vary in discriminability. Thus, there is some correspondence between judgements of distinctiveness and search RTs or fixation durations, but the correspondence is patchy.

### Conclusions

We evaluated whether there was an automatic tendency to look longer at attractive faces in a visual search task. Visual search tasks have a high ecological validity because they correspond to a frequent everyday activity. In two experiments, we varied the implicit search intentions of the participants by changing the search target. When participants searched for disgusted or sad faces, fixation durations decreased with attractiveness. The most likely reason is that disgusted or sad faces are perceived as less attractive than neutral faces, which created an implicit intention to look for unattractive faces. Conversely, search for happy faces created the implicit intention to look for attractive faces and fixation durations increased with attractiveness. Thus, the relation between fixation durations and attractiveness depended on the search task and fixation durations were not automatically longer for attractive faces. Our results do not challenge a basic tenet of evolutionary psychology, the attunement to relevant information in the environment, because increased attention to beauty does occur, but not with conflicting task demands.
